# Criterion validity of a 10-category scale for ranking physical activity in Norwegian women

**DOI:** 10.1186/1479-5868-9-2

**Published:** 2012-01-19

**Authors:** Kristin B Borch, Ulf Ekelund, Søren Brage, Eiliv Lund

**Affiliations:** 1 Department of Community Medicine, University of Tromsø, NORWAY; 2 Medical Research Council Epidemiology Unit, Institute of Metabolic Science, Cambridge, England, UK

**Keywords:** Accelerometry, combined sensor, heart rate, physical activity, self-report, validation

## Abstract

**Background:**

Accurate measurement of physical activity (PA) is critical to establish dose-response relationships with various health outcomes. We compared the self-administered PA questionnaire from the Norwegian Women and Cancer Study (NOWAC) with a criterion method in middle-aged Norwegian women.

**Methods:**

A sample of 177 randomly recruited healthy women attended two clinical visits approximately 4-6 months apart. At each visit, the women completed the NOWAC PA questionnaire (NOPAQ), rating their overall PA level on a 10-category scale (1 being a *"very low" *and 10 being a *"very high" *PA level) and performed an 8-minute step-test to estimate aerobic fitness (VO_2_max). After each visit, the women wore a combined heart rate and movement sensor for 4 consecutive days of free-living. Measures of PA obtained from the combined heart rate and movement sensor, which were used as criterion, included individually calibrated PA energy expenditure (PAEE), acceleration, and hours/day of moderate-to-vigorous intensity PA (MVPA). These were averaged between visits and compared to NOPAQ scores at visit 2.

**Results:**

Intra-class correlation coefficients for objective measures from both free-living periods were in the range of 0.65-0.87 (*P *< 0.001), compared to 0.62 (*P *< 0.001) for NOPAQ. There was a moderate but significant (*P *< 0.001) Spearman's rank correlation coefficient in the range of 0.36-0.46 between NOPAQ and objective measures of PA. Linear trends for the association between the NOPAQ rating scale with PAEE, hours/day of MVPA and VO_2_max *(P *< 0.001) were also demonstrated.

**Conclusions:**

Self-reported PA level measured on a 10-category scale appears valid to rank PA in a female Norwegian population.

## Background

In large-scale epidemiologic studies, physical activity (PA) is often assessed using questionnaires [1,2]. Self-report methods as global questionnaires are commonly used to assess the relationship with health outcomes in order to rank or classify individuals as either physically active or inactive [3,4]. Indeed, a number of different PA questionnaires have been developed for various purposes such as surveillance, etiological investigation and risk stratification [5]. PA is a complex multidimensional behavior characterized in terms of volume, domain, type, duration, intensity and frequency [6], which makes PA inherently difficult to assess accurately in epidemiologic studies. Particularly challenging is the estimation of PA energy expenditure (PAEE) [4,7]. Nonetheless, documentation of a questionnaire's precision is important to interpret the information it provides. Independent criterion methods that can accurately assess PAEE is key when examining the validity of PA questionnaires that aim to estimate PAEE as an integrated measure of self-reported duration, intensity and frequency of PA [3]. The gold standard for measuring PAEE during free-living conditions is the doubly-labeled water method, combined with an assessment of resting metabolic rate. However, this approach is expensive and does not provide any information on intensity and frequency patterns. Therefore, the most commonly used measures are derived from accelerometry, heart rate monitoring, or combined heart rate and movement sensing.

The Norwegian Women and Cancer (NOWAC) Study is a population-based, nation-wide cohort study that was initiated in 1991 [8]. The study was originally set up to estimate the risk of breast cancer and its association with oral contraceptive use, and has since been expanded to examine the possible association between several exposures and different types of cancer and other chronic diseases [8]. The NOWAC PA questionnaire (NOPAQ) is a unique and simple self-report instrument for ranking of PA level using a 10-category scale. It was mainly developed to enable adjustment for PA as a confounding variable when examining associations between other health-related exposures and disease. PA exposure as assessed by the NOPAQ has not been previously validated. We therefore aimed to evaluate the criterion-related validity of the NOPAQ against objective estimates of PA, as assessed by individually-calibrated combined heart rate (HR) and movement sensoring. In addition, we examined the influence of aerobic fitness (VO_2_max). The results from this study will provide useful knowledge regarding PA assessment methodology within a female population.

## Methods

### Study Design

The study was carried out from March 2007 to March 2008, covering all four seasons. It consisted of two clinical visits, approximately 4 to 6 months apart (mean time between visits = 4.99 (0.92 months). Each clinical visit included completion of the NOPAQ and an 8-minute step-test. Each clinical visit was followed by combined heart rate (HR) and movement monitoring for at least 4 consecutive days and nights of free-living. This study followed the protocol of The InterAct validation study [9], which aimed to validate different PA questionnaires within the European Prospective Investigation into Nutrition and Cancer, of which the NOWAC study is part.

### Study population

**Figure 1:**
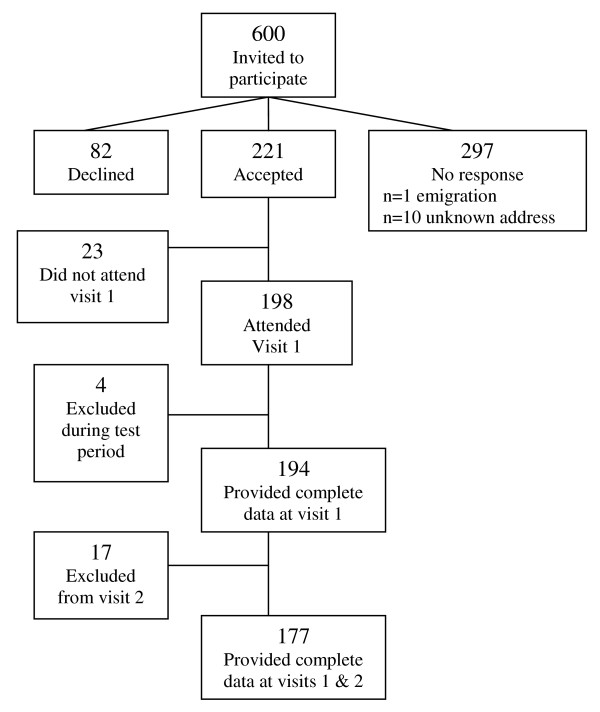
**Flow chart - inclusion of the study participants**.

Tromsø is a city located at latitude 69°N in the county of Troms, an area characterized by large seasonal variations. A random sample of 600 women between 40-55 years of age and living in Tromsø was drawn in 2007 from the National Population Registry, Statistics Norway. Due to emigration and unknown addresses, 589 women were invited to participate in this study. The participants had to live in the same municipality as the investigation premises at the University of Tromsø to match the original sample in the NOWAC study [8]. Exclusion criteria were conditions that had led to mobility limitations, which made walking unaided impossible. A total of 221 women agreed to participate; however 23 women did not come to the first clinical visit, resulting in an initial study sample of 198 women (overall response rate 33.6%). Following the two clinical visits complete data was available for 177 women: 4 did not provide sufficient free-living data at visit 1 and a further 17 had missing data at visit 2 (Figure 1). Participants taking medications that affected heart rate (use of beta blockers, 50% or more of maximum dose, n = 1) were excluded from the step-test, but were included in all other parts of the study. Written informed consent was obtained from each participant and ethical approval for the study was obtained from the Regional Committee for Medical Research Ethics, North of Norway, and the Norwegian Data Inspectorate.

### Anthropometrics

Height (to the nearest mm) and weight (to the nearest 0.1 kg) were measured by a Seca 764 electronic body height and weight measuring instrument (Seca gmbh & co.kg, Hamburg, Germany). Both were measured with the participant in light clothing and without shoes. Body mass index was calculated as weight in kilograms divided by height in meters squared (kg/m^2^), and categorized into three levels; normal (< 25 kg/m^2^), overweight (25-30 kg/m^2^) and obese (> 30 kg/m^2^).

### Measures of PA

#### Objective measures of PA and fitness

Eligible participants were fitted with a combined heart rate and movement sensor (Actiheart, CamNtech, Cambridge, UK), which was attached to the chest via two standard electrocardiogram electrodes [10].

Study participants performed an 8-minute ramped step-test using a 200-mm step (Reebok, Lancaster, UK) to determine the individual relationship between heart rate and workload [11]. Specifically, participants were asked to step up and down following a timed voice prompt at a step frequency that began at 15 body lifts per minute (60 steps/min) and increased linearly to a maximum of 33 lifts per minute, immediately followed by a 2-minute seated recovery phase. In addition to their utility for individual calibration of free-living heart rate data, the parameters of the step test were used to estimate VO_2_max (mL/kg/min) by extrapolating each individual's sub-maximal HR-PAEE relationship to age-predicted maximum HR [12] and adding an estimate of resting metabolic rate [13].

Following each step-test, the combined heart rate and movement sensor was downloaded and re-initialized for long-term recording on a 1-minute epoch and participants were instructed to wear the monitor continuously for a minimum of 4 consecutive days and nights. Data collected during these free-living periods was downloaded to a computer database and the heart rate trace was processed using a robust Gaussian Process regression method to handle potential measurement noise [14]. PA intensity (J/min/kg) for each time point was estimated from the combination of individually calibrated heart rate and movement data [11] using a branched equation framework [15]. Periods of non-wear were inferred from the combination of non-physiological heart rate and prolonged periods of inactivity, which were taken into account to minimize diurnal information bias when summarizing the intensity time-series into PAEE (kJ/kg/day), hours per day of light intensity PA (< 3 metabolic equivalent of task units, MET), moderate-to-vigorous PA (MVPA, > 3 MET), and average acceleration (m/s^2^).

#### PA questionnaire (NOPAQ)

Study participants completed the NOPAQ at both clinical visits. The questionnaire asks the participant to rate their PA level at age 14 and 30 years, as well as "today" (the time of completion) on a scale of 1 to 10, where 1 corresponds to a *"very low" *PA level and 10 corresponds to a *"very high" *PA level. PA was described in the questionnaire as *"By physical activity we mean both work in and outside the home, as well as training/exercise and other physical activity, such as walking, etc. Mark the number that best describes your level of physical activity." *(See additional file 1 showing questionnaire excerpt). The response given for the PA level at the time of questionnaire completion ("today") was evaluated in this validation study.

### Statistical analysis

Descriptive data (age, height, weight, body mass index, fitness, and objective physical activity measures) were first summarized on an individual level based on measures from both clinical visits and the sample mean ± standard deviation (SD) was then reported. Outcomes from the NOPAQ were described with frequency distribution. Responses from the NOPAQ completed at visit 2 were used in the comparison with objective measures. Relative agreement between visit 1 and visit 2 measures were examined by Intra-class correlation coefficients (ICC). Time between assessments did not influence these results, so they were left unadjusted. Criterion validity of the NOPAQ against objective measures (PAEE, accelerometry, MVPA and VO_2_max) was examined by Spearman's rank correlation coefficient (σ). The NOPAQ was analyzed using each level of the 10-category scale as a categorical variable in correlation analyses. Since participants may also take their perceived fitness level into account when self-reporting their PA level, we also combined the VO_2_max estimate and the PAEE estimate into a new variable by averaging their z-scores (zPAFIT), and compared with the NOPAQ. Linear regression analysis was used to examine relationships between the PAEE and zPAFIT measures and the NOPAQ (self-reported PA level treated as a continuous variable) at three levels of adjustment; unadjusted, age and BMI adjusted, and with additional adjustment for VO2max (PAEE model only). Assumptions of linear regression analysis (i.e., normally distributed residuals) was examined by the Shapiro-Wilk W test. For all analyses, the level of significance was set at *P *< 0.05. STATA version 11.0, special edition (StataCorp, College Station, TX, USA) was used for the statistical analyses.

## Results

### Characteristics of the study participants and PA level

Table 1 presents descriptive characteristics of study participants. The median self-reported PA level was 5 on the 10-category scale, with 41.2% of participants ranking their PA level as 5 or 6, 28.3% as 1 to 4 and 30.5% ranking their PA level as 7 or higher. The participants spent on average 41% of their time at sedentary (≤ 1 MET) and 53% at low (1-3 METs) PA intensity according to combined sensor measures of PA. The monitor was worn by participants for an average of 4.7 (0.22) days and nights during each of the two measurement periods.

**Table 1:** Descriptive characteristics of study participants, n = 177 women.

Characteristics	Mean	SD	Min	Max
**Age (years)**	47.5	4.4	40	55
**Height (m)**	1.7	0.06	1.51	1.8
**Weight (kg)**	71.1	10.9	47	115.3
**BMI (kg/m^2^)**	26.1	3.5	18.8	37.5
**PAEE (kJ/kg/day)**	44.9	14.4	17.5	96.6
**Acceleration (m/s^2^)**	0.1	0.04	0.02	0.3
**Sedentary time (hours/day)**	9.9	1.4	6.6	15.4
**Low intensity PA (hours/day)**	12.6	1.4	7.5	16.0
**MVPA (hours/day)**	1.5	0.9	0.2	6.5
**VO_2_max (mlO_2_/min/kg)**	31.9	4.8	22.8	53.9
NOPAQ*	5	3	1	10

*Self-reported PA level median (interquartile range)

Table 2 displays the ICC for PAEE, acceleration, MVPA, VO_2_max, zPAFIT and the NOPAQ (all P < 0.001). It indicates a moderate agreement for all activity measures, and similar between objective and self-report measures. VO_2_max measures at first and second clinical visit were more strongly correlated, with an ICC of 0.87.

**Table 2:** Relative agreement between measures obtained from the two visits

Outcome	ICC*	95% CI^†^
**PAEE**	0.65	[0.56, 0.74]
**MVPA**	0.66	[0.56, 0.74]
**Acceleration**	0.65	[0.56, 0.73]
**VO_2_max**	0.87	[0.81, 0.89]
**zPAFIT**	0.84	[0.79, 0.88]
**NOPAQ**	0.62	[0.52, 0.71]

*Intraclass correlation coefficient

^†^Confidence interval

### Comparing self-reported PA level and objective measures

Sensor-based measures of PA and fitness were stratified by self-reported PA levels (Table 3). Overall, significant linear trends of PAEE, acceleration, MVPA, VO_2_max and zPAFIT across self-reported PA levels were observed. Self-reported PA level was positively associated with level of PAEE, and a trend of increasing acceleration from the lowest to the highest self-reported PA level was also observed. Higher self-reported PA level was associated with higher durations of MVPA (> 3 METs). Similarly, those who characterized themselves as less active accumulated less MVPA. Furthermore, higher self-reported PA levels were associated with higher VO_2_max estimates and lower body mass index.

**Table 3:** Objective measures of physical activity and fitness by self-reported PA level among 177 women.

PA level	% PA (N)	PAEE	MVPA	Acceleration	VO_2_max	zPAFIT	BMI
**1**	1.7 (3)	31.8 (4.50)	.80 (.10)	.07 (.003)	29.1(1.10)	-.70 (.33)	29.1(2.68)
**2**	5.1 (9)	35.7 (3.17)	.87 (.19)	.08 (.006)	29.9 (.95)	-.42 (.19)	25.0(1.16)
**3**	7.4 (13)	37.7 (5.01)	1.16 (.31)	.08 (.006)	22.4 (.89)	-.59 (.27)	27.5(.96)
**4**	14.1(25)	39.3 (2.07)	1.31 (.15)	.09 (.007)	29.9 (.67)	-.43 (.11)	27.4 (.72)
**5**	23.2(41)	42.4 (1.97)	1.38 (.10)	.11 (.006)	31.6 (.91)	-.13(.15)	26.7 (.60)
**6**	18.1(32)	47.7 (2.62)	1.49 (.13)	.13 (.009)	33.4 (.65)	.35 (.13)	24.1 (.53)
**7**	17.5(31)	52.1 (2.43)	1.98 (.15)	.12 (.006)	33.8 (.84)	.32 (.14)	26.4 (.60)
**8**	10.2(18)	47.7 (2.39)	1.67 (.14)	.14 (.008)	32.3 (.70)	.10 (.12)	25.7 (.63)
**9**	1.1 (2)	57.1(17.91)	2.44(.1.30)	.13 (.01)	29.7(3.72)	.12 (.81)	25.0 (.81)
**10**	1.7 (3)	70.4(14.40)	3.42 (1.58)	.16 (.04)	41.1(7.15)	1.84(1.14)	25.1(1.86)

Test for trend		*P *< 0.001	*P *< 0.001	*P *< 0.001	*P *< 0.001	*P *< 0.001	*P *= 0.047

The associations between the NOPAQ and criterion measures were significantly correlated, with Spearman's rank correlation coefficients of 0.36-0.46, depending on the criterion measure: PAEE (σ = 0.39), MVPA (σ = 0.42), VO_2_max (σ = 0.36) and zPAFIT (σ = 0.41). The strongest correlation was observed for accelerometry (σ = 0.46).

Relationships between the NOPAQ and the criterion measures PAEE and zPAFIT were examined by linear regression analysis (Table 4). Self-reported PA level contributed significantly to explain variance in both PAEE and zPAFIT at all levels of adjustment.

**Table 4:** Relationship between self-report and objective measures of physical activity

	PAEE			zPAFIT		
		
Model	β	95% CI	R2	β	95% CI	R2
**NOPAQ**	0.049	[0.029, 0.069]	0.12	0.18	[0.12, 0.26]	0.15
**NOPAQ** *(w/BMI and age)*	0.039	[0.021, 0.056]	0.34	0.15	[0.09, 0.21]	0.34
**NOPAQ** *(w/BMI, age, and VO_2_max)*	0.021	[0.005, 0.038]	0.48			

The β-coefficient denotes the difference in the outcome variable by a 1-unit difference in the NOPAQ scale. CI = Confidence Interval. R2 = model explained variance

## Discussion

The present study examined the criterion validity of a simple self-reported ranking instrument, the NOPAQ, which was aimed at ranking overall PA level in Norwegian women aged 40-55 years. In general, the results showed significant but moderate Spearman's rank correlation coefficients in the range of 0.36-0.46, and linear trends in the relationship between self-reported PA level and objective measures of PA derived from registration of heart rate and movement during free-living, including PAEE, acceleration, and MVPA. In addition, NOPAQ correlates positively with VO_2_max. The results also showed that stability statistics were comparable for both objective and subjective activity measures over a period of 4-6 months, as assessed using ICCs (0.62-0.65). Taking into account that PA is a modifiable behavior, this indicates that physical activity level of middle-aged women may be relatively stable over a time period of 4 to 6 months, and across large seasonal variations in Norway, although not as stable as fitness.

Our results are in agreement with previous studies examining the validity of other self-reported ranking instruments to measure global PA. In a review [16] on self-reported PA questionnaires comparing different objective measures of PA (e.g., doubly-labeled water method, accelerometry, and HR monitoring) with self-reported PA, the correlation coefficients ranged between 0.14-0.36. More complex questionnaires have been compared with relatively simple measures of global PA and correlation coefficients were reported in the range of 0.14-0.41 [17]. Long and complex PA questionnaires may be demanding for the respondents and may therefore explain why some of the highest coefficients for reliability and validity are seen for global PA questionnaires [17]. A validation study of the International Physical Activity Questionnaire against accelerometry across 12 countries reported a criterion validity by Spearman correlation with a median of 0.30 [18]. It is interesting that the simple NOPAQ ranking instrument correlates quite strongly with the criterion measures, even higher than some PA questionnaires that aim to measure different dimensions of PA. However, an alternative explanation is that the lower correlations observed in other studies are due to limitations of the criterion method used for comparison; even though the correlation with accelerometry in our study was also high, this criterion measure may have been stronger in our study since the participants wore the monitor 24 hours a day during two 4-day observation periods. We cannot however compare correlations directly to ascertain which self-reported instrument may be superior. One exception is the recent report by the InterAct Consortium on validity of the short EPIC questionnaire, carried out in 10 European countries and including the present sample of Norwegian women [9]. This study, using the same criterion measures and sampling protocols, reported Spearman correlations of 0.32 and 0.35 in the Norwegian sample for the best indices derived from that questionnaire, which is almost identical to the NOPAQ result. The acceptable measurement properties of the NOPAQ compared to other more established self-reported instruments could be due to the simplicity of its design. It may be easier for the participant to answer questions on PA when only one general dimension of PA is sought [19]. The NOPAQ includes several dimensions collapsed into one global PA measure, and could be considered beneficial. To this end, face validity of the NOPAQ was recently demonstrated in a follow-up within the original NOWAC study, in that PA level was predictive of mortality and also inversely associated with BMI [20].

Strengths of the present study include the criterion method of individually calibrated HR and movement sensing in a repeated measurement study design. Using multiple measures will most likely provide a more robust estimate of "true" PA, as compared to using a single measure. Apart from the doubly-labeled water method, combined HR and movement sensoring is probably the most accurate criterion method available from which to derive PAEE measures [5]. Indeed, a combined sensor that provides estimates of PAEE using accelerometry and heart rate monitoring data may overcome some of the limitations associated using these methods separately. Accelerometry, for example, is limited for assessing PAEE of certain types of activities such as cycling and carrying heavy loads. Heart rate monitoring is limited when measuring low-intensity activity due to stronger relative influence of stress and relies more heavily on individual calibration [15]. A combined monitor with individual calibration of the heart rate-PAEE relationship and branched modeling for combining the physiological and biomechanical information recorded has been shown to reduce the error of predicted PAEE [11]. The criterion measure of PA intensity and its time-integral, PAEE, has been successfully validated against indirect calorimetry during simulated daily living activities [21-23] and during free-living against the doubly-labeled water method [24].

Between 3 and 5 days is usually considered appropriate for providing reliable measures of free-living PA by accelerometry [25]. In this study, the participants wore the monitors for > 4 days including nights after each clinical visit, which is also considered sufficient for obtaining reliable PAEE estimates. This protocol also provided data on sleeping heart rate which, in addition to aiding individual calibration, improved the precision of PAEE estimates [15]. Another important strength of this study includes a 24-hour protocol which makes non-wear time less of an issue. The monitors were given to participants during a face-to-face clinical visit and this approach made certain that care and use of the monitor was explained adequately.

Our results also indicated that higher VO_2_max was associated with higher self-reported PA level. It is a general understanding among many people that fitness (VO_2_max) is the same as PA [26], despite the fact that the constructs are different, one being a capacity ("can do") quantity and the other being a behavioral ("will do") quantity. It is also argued that most people have a fairly clear perception of their fitness level according to their leisure time PA levels [17] but to generalize such predictions is perhaps questionable. To correct for the possibility that participants may take into account their perceived fitness level when reporting their PA level and the resulting bias, the association with PAEE was adjusted for VO_2_max in the linear regression analysis, in addition to examining a composite outcome of PAEE and VO_2_max in the zPAFIT variable. The magnitude of the association between NOPAQ and PAEE was attenuated but still significant after adjusting for fitness. In addition, the zPAFIT variable showed only minor improvement in the correlation with self-reported PA level, which suggests that the NOPAQ does not suffer severely from any bias by VO_2_max level.

The participants in the present study were representative of the population of the NOWAC Study in terms of age and sex. The NOWAC study population is further considered representative of Norwegian middle-aged women [8]. Nonetheless, it is important to highlight that examining agreement between instruments done in one group may not generalize to others. The NOPAQ was completed twice, at two separate clinical visits by participants in this study, and an ICC of 0.62 indicated a moderate agreement; the responses did not differ substantially over a 4-6 month time period. The participants live in an area with considerable seasonal variations, which could explain some of the differences in self-reported PA level between visits. This is supported by the fact that ICCs were similar for sensor-based measures of PA.

The main advantages of a PA questionnaire such as the NOPAQ that uses an ordinal scale are the ease of design, administration, and data handling. Nevertheless, there are other challenges associated with the use of a simple rating scale. Studies have demonstrated that self-reported PA is usually overestimated when compared with criterion measures of PA [27,28]. Calibrating highly subjective experiences such as perceived total amount of PA along a continuum of equal intervals is difficult. Assessment of a subjective phenomenon will always be a challenge considering the fact that categorizing and quantification never will totally embrace a phenomenon [29]. Another criticism of an ordinal scale is the reluctance of subjects to make full use of its range, preferring to avoid extreme responses [30]. A numeric scale will obviously raise difficulties for some individuals in rating a self-perceived behavior like PA, and reference frames will probably differ widely. In order to use a scale well, respondents need a reference frame when choosing PA levels [31]. The challenge with the NOPAQ is that it uses a numeric scale to rank PA levels in individuals. It could be difficult for the respondent to differentiate between and interpret the 10 levels without a reference frame to associate with the various levels of the scale. Both vigorous PA and sedentary behavior are two important sub-dimensions of PA related to health [32]. Our results suggest that the NOPAQ is sufficient to rank individuals into categories of global PA level which is of relevant usability. However, the scale does not give insight into the type, frequency, intensity, or duration of PA, nor the domains in which PA takes place; these characteristics limits the utility of the NOPAQ. A limitation of the present study was that there were fewer individuals in the lower and higher self-reported PA categories. However, we would not expect many women between 40 and 55 years to be categorized into either of these groups, and we found the study population normally distributed according to self-reported PA level.

The fact that correlation coefficients are significant does not automatically imply that the two measures are identical and the results should be interpreted accordingly. Both questionnaire- and sensor-based measures of activity clearly assess PA differently, each with individual strengths and limitations.

It is important to maintain consistency in surveillance of PA in a prospective cohort study like the NOWAC Study, and proceed with the same PA questionnaire for future follow-up. However, one should consider an alternative PA questionnaire when it comes to quantifying PA level in different domains and types, as well as the duration, intensity and frequency that make up the total volume of PA and help to understand the mechanisms of PA and its relation to different diseases. As for the continued use of the NOPAQ in the NOWAC Study, results from our present report are encouraging and demonstrate the criterion-related validity of the measure used to define PA level in this population.

## Conclusions

In summary, the NOPAQ was found to be valid, with modest correlations compared with criterion measures, making this self-report instrument suitable to differentiate general PA levels in an adult female population in Norway.

## Competing interests

The authors declare that they have no competing interests.

## Authors' contributions

KBB collected data, carried out the statistical analysis and drafted the manuscript. UE has made substantial contributions to conception and design and has contributed with critical revision of the manuscript. SB designed and derived all objective measures of activity and fitness, contributed to the statistical analysis and interpretation of the data and critical revision of the manuscript. EL is the principal investigator and designed the NOWAC Study, and contributed with critical revision of the manuscript. All authors read and approved the final manuscript.

## Supplementary Material

Additional file 1 **The Norwegian Women and Cancer Study questionnaire on physical activity (NOPAQ)**. Click here for file
