# Supplementary material for: Criterion validity of a 10-category scale for ranking physical activity in Norwegian women
Source: Int J Behav Nutr Phys Act. 2012 Jan 19;9:2. doi: 10.1186/1479-5868-9-2 (PMC3398334; doi:10.1186/1479-5868-9-2)
Supplement: Additional file 1 — The Norwegian Women and Cancer Study questionnaire on physical activity (NOPAQ). [file 1479-5868-9-2-S1.PDF]

Physical activity

Please indicate the level of your physical activity on a scale from very low to very high (at the ages of 14 and 30 years, and today). The scale below goes from 1-10. By physical activity we mean both work in and outside the home, as well as training/exercise and other physical activity, such as walking, etc. Mark the number that best describes your level of physical activity

Age	Very low									Very high
At 14 years	1	2	3	4	5	6	7	8	9	10
At 30 years	1	2	3	4	5	6	7	8	9	10
Today	1	2	3	4	5	6	7	8	9	10
